# Stroke Induces a BDNF-Dependent Improvement in Cognitive Flexibility in Aged Mice

**DOI:** 10.1155/2019/1460890

**Published:** 2019-05-05

**Authors:** Josh Houlton, Lisa Y. Y. Zhou, Deanna Barwick, Emma K. Gowing, Andrew N. Clarkson

**Affiliations:** Department of Anatomy, Brain Health Research Centre and Brain Research New Zealand, University of Otago, Dunedin 9054, New Zealand

## Abstract

Stroke remains a leading cause of disability worldwide. Recently, we have established an animal model of stroke that results in delayed impairment in spatial memory, allowing us to better investigate cognitive deficits. Young and aged brains show different recovery profiles after stroke; therefore, we assessed aged-related differences in poststroke cognition. As neurotrophic support diminishes with age, we also investigated the involvement of brain-derived neurotrophic factor (BDNF) in these differences. Young (3-6 months old) and aged (16-21 months old) mice were trained in operant touchscreen chambers to complete a visual pairwise discrimination (VD) task. Stroke or sham surgery was induced using the photothrombotic model to induce a bilateral prefrontal cortex stroke. Five days poststroke, an additional cohort of aged stroke animals were treated with intracerebral hydrogels loaded with the BDNF decoy, TrkB-Fc. Following treatment, animals underwent the reversal and rereversal task to identify stroke-induced cognitive deficits at days 17 and 37 poststroke, respectively. Assessment of sham animals using Cox regression and log-rank analyses showed aged mice exhibit an increased impairment on VD reversal and rereversal learning compared to young controls. Stroke to young mice revealed no impairment on either task. In contrast, stroke to aged mice facilitated a significant improvement in reversal learning, which was dampened in the presence of the BDNF decoy, TrkB-Fc. In addition, aged stroke control animals required significantly less consecutive days and correction trials to master the reversal task, relative to aged shams, an effect dampened by TrkB-Fc. Our findings support age-related differences in recovery of cognitive function after stroke. Interestingly, aged stroke animals outperformed their sham counterparts, suggesting reopening of a critical window for recovery that is being mediated by BDNF.

## 1. Introduction

Poststroke disability can include impairments in motor, sensory, visual, and cognitive functions [1]. Cognitive impairments, like motor impairments, can persist for years, leading to increased burden on caregivers and society [2, 3]. An added complication of cognitive impairments is that epidemiological evidence shows that impairments arising from strokes to the prefrontal cortex (PFC) or parietal cortex can take several months before becoming apparent [2, 4–6]. Whilst cognitive impairments are present in the traditional middle cerebral artery occlusion models of stroke, cognitive assessment in these models are often confounded by the presence of gross motor impairments that are required to be intact in order to complete the cognitive tasks themselves [7]. Furthermore, our knowledge of the mechanisms that underlie cognitive impairments following stroke remains inadequate and additional research is still required to determine which intervention to use and at what time point should treatment begin.

In an effort to assess changes in cognition following stroke, several groups have established stroke models targeting the PFC, reporting deficits in spatial memory and executive function in the absence of motor impairment [8–10]. The rationale for targeting the PFC is that it is one of several key areas involved in higher order cognitive processing, such as executive function, attention, behavioural inhibition, and goal-directed learning [11, 12]. In addition, the PFC region is linked with normal age-related cognitive decline, as well as behavioural impairments in neurodegenerative disorders in both rodents and humans [13–15]. As many as 92% of stroke survivors report some form of cognitive decline, including impairments in attention, working memory, and executive function, which includes cognitive flexibility [16, 17]. Cognitive flexibility is what allows one to adapt to new and unexpected conditions in our day-to-day lives; without it, even the smallest of tasks would become a huge ordeal.

Preclinical assessment of cognitive impairments is limited by the absence of tests that are considered to be translational. This, however, has changed in recent years with the development of touchscreen-based cognitive testing for rodents that allow us to assess components of human-based cognition which are assessed using the Cambridge Neuropsychological Test Automated Battery (CANTAB) assessment tools [18–21]. Importantly, various behavioural tests have been developed to assess cognitive impairments linked to disease-based genetic mutations using identical paradigms in both humans and rodents [18, 19]. In addition, lesions to the medial PFC (mPFC) have been shown to play a role in impaired reversal learning, specifically when rodents are presented with complex images using touchscreens [22, 23].

Given the translatability of the touchscreen technology, we aimed to further characterise our PFC stroke model to see if this extends to impaired cognitive flexibility as assessed using the visual discrimination (VD), reversal, and rereversal tasks. As 75-89% of all strokes occur in people aged 65 and over [24], we also aimed to assess how aged mice would perform on this task. Moreover, neurotrophins such as brain-derived neurotrophic factor (BDNF), which play an important role in regulating plasticity, have been shown to diminish with age [25, 26]. Therefore, we also investigated the involvement of BDNF in the poststroke recovery of cognitive function as we and others have previously reported this neurotrophin to be critical for poststroke recovery of motor function [27–30].

## 2. Methods

### 2.1. Animals and Surgical Procedures

All procedures described in this study were carried out in accordance with the guidelines on the care and use of laboratory animals set out by the University of Otago, Animal Research Committee and the Guide for Care and Use of Laboratory Animals (NIH Publication No. 85-23, 1996). Mice were housed under a 12-hour light/dark cycle with ad libitum access to food and water. All young animals were paired based on initial learning rates on the VD task and then randomly assigned to either sham or stroke surgery. Aged animals were also grouped based on their initial learning rate and randomly assigned to either sham, stroke, or stroke+TrkB-Fc treatments. All assessments were carried-out by observers blind as to the treatment group. For the initial experiment comparing the effect of age and stroke, focal stroke to the PFC of young (3-6 months old, *n* = 13) and aged (16-21 months old, *n* = 10) male C57BL/6J mice was induced by photothrombosis as previously described [10]. Under isoflurane anaesthesia (2-2.5% in O_2_), mice were placed in a stereotactic apparatus and the skull was exposed through a midline incision, cleared of connective tissue, and dried. A cold light source (KL1500 LCD, Zeiss) attached to a 40x objective giving a 2 mm diameter illumination was positioned 1.2 mm anterior to Bregma, and 0.2 mL of Rose Bengal solution (Sigma-Aldrich; 10 g/L in normal saline) was administered through intraperitoneal (i.p.) injection. After five minutes, the brain was illuminated through the intact skull for 22 minutes, creating bilateral lesions to the PFC [10]. Young (*n* = 13) and aged (*n* = 9) sham animals received the same surgery as above, with a 0.2 mL injection of saline (i.p.) instead of Rose Bengal. Another cohort of aged mice (16-21 months old) also received the above stroke surgery prior to TrkB-Fc (*n* = 16) or IgG-Fc (*n* = 10) hydrogel administration, as described in Section 2.2.

Data obtained from animals in the study were collected across three cohorts due to the limited availability of the aged animals. We tried to keep treatment group size consistent across these cohorts; however, we noted the high attrition and mortality rates of our aged animals in some groupings, in particular the TrkB-Fc cohort. Therefore, some of the sham animals were reallocated and used in the stroke cohorts. A summary of the initial group sizes and number of mortalities between treatment groups can be seen in Table 1.

### 2.2. In Vivo Drug Dosing

A hyaluronan/heparan sulfate proteoglycan biopolymer hydrogel (HyStem-C, BioTime Inc., Alameda, CA) was employed to locally deliver TrkB-Fc or human IgG-Fc (antibody and vehicle control) five days poststroke to the peri-infarct cortex as described previously [27, 31].The timinign of treatment fits with the critical period when we know plasticity exists, that is, 3-14-days poststroke onset [32]. The stroke core is fully formed by 3 days, and we and others have shown that starting treatments 3-5 days poststroke does not interfere with the stroke itself but changes the state of plasticity and improves functional recovery, including treatments that alter BDNF and TrKB signalling [27, 28, 33]. Therefore, consistent with previous studies we administered TrkB-Fc or IgG-Fc five days poststroke. Following stroke, BDNF expression remains elevated for at least 3 weeks [27, 33, 34]. Similar profiles have been detected when using hydrogel delivery systems, with reports of small peptides (including TrkB-Fc) still being released 4 weeks after administration [25, 29, 30]. Therefore, hydrogel administration from 5 days poststroke would still be releasing TrkB-Fc or IgG-Fc at the start of the rereversal period, thereby inhibiting the later stroke-induced BDNF response and later BDNF-mediated learning.

A total of 7.5 *μ*L of HyStem-C was impregnated with either TrkB-Fc (5 *μ*g/mL) or human IgG-Fc (5 *μ*g/mL). HyStem-C was prepared according to the manufacturer's instructions. In brief, TrkB-Fc or human IgG-Fc was added to the HyStem/Gelin-S mix (component 1 of hydrogel), followed by the addition of Extralink (component 2 of the hydrogel) in a 4 : 1 ratio. The impregnated HyStem-C mix was injected immediately after preparation into the stroke cavity using a 30-gauge needle attached to a Hamilton syringe at stereotaxic coordinates 1.2 mm AP, 0 mm ML, and 0.75 mm DV.

### 2.3. Behavioural Assessment

The VD task has previously been used to identify an animal's perceptual ability, as well as testing their performance on an associative learning task [20]. Often, VD is paired with the reversal learning task in order to test behavioural flexibility, which can be disrupted as a consequence of have a neurological condition [20, 22].

Initial protocols for VD and reversal learning were adapted from those first described by Mar et al. and Brigman and Rothblat [21, 22]. Mice were trained to discriminate between two images, a solid white flash or wheel, presented in a spatially pseudorandomised manner, across a maximum of 30 trials per session. The two images were chosen following personal communication with a representative from Campden Instruments Ltd. (Julie Gill, personal communication, 2014), who observed equal salience following the presentation of both stimuli, which has been recently validated in environmentally enriched animals [35]. Animals were randomly allocated to learn either the flash or wheel stimuli during the initial VD learning task. When we established the randomisation, we also ensured that stimuli allocation was balanced between testing chambers and sequential runs. Correct responses for one stimulus resulted in an audio tone and reward (*S*+, correct), whilst responses for the other stimulus resulted in no reward (*S*−, incorrect) and a five second “time-out” period, during which time the house light was turned on. Incorrect responses were followed by a correction trial, where the same image was repeatedly presented in the same position, until a correct response was made.

**Figure 1:**
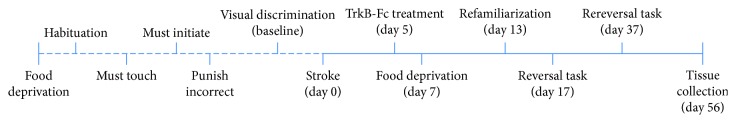
Experimental timeline illustrating the sequence of events in the current experiment. The dotted line represents pretraining prior to stroke surgery. The solid line refers to postsurgery events, with reference to the number of days after stroke.

Acquisition criterion for the VD task was ≥80% correct responses across 30 trials within 60 minutes, with the criterion needing to be achieved over two consecutive days. Once the criterion was reached, animals were matched into groups based on initial learning rates (days to acquisition) before being randomly allocated to one of the treatment groups. Following surgery, animals were allowed up to a week to recover before undergoing food deprivation, refamiliarization on the original VD stimuli for four sessions, and then beginning the reversal task at 17 days poststroke. In this task, the stimulus that previously elicited a reward becomes nonrewarded, and vice versa (*S*+→*S*−). Lastly, all animals then underwent the rereversal task at 3 days poststroke, where the correct and incorrect stimuli were switched back to their original responses. The criterion for the reversal and rereversal task was the same as the VD criterion. Animals were tested daily until criterion was met for all tasks. This experimental timeline is shown diagrammatically in Figure 1.

The data obtained for the VD and both the reversal and rereversal tasks include the number of consecutive days required to reach criteria, total number of trials to reach criteria, total number of correction trials made to reach criteria, and total intertrial interval (ITI) touches to make criteria.

### 2.4. Infarct Volume

At the completion of the VD task, animals were anesthetised and transcardially perfused with 4% paraformaldehyde (PFA). The brains were removed and postfixed for 1 hour in 4% PFA before being transferred to 30% sucrose. The brains were cut coronally with a section thickness of 40 *μ*m on a sliding microtome with a freezing stage, with all sections stored in cryoprotectant at -20°C. Infarct volume was determined by histological assessment using a previously published cresyl violet staining protocol [36]. Infarct volume was quantified using ImageJ (National Institutes of Health, USA) by an observer blind as to the treatment groups and was based on obtaining measurements from every 6th section through the entire infarct (area in mm^2^), with infarct volume being quantified as follows:

(1) $$\begin{array}{c} \text{infarct volume }\left({\text{mm}}^{3}\right)=\left(\text{area }\left({\text{mm}}^{2}\right)\times \text{section thickness}\times \text{section interval}\right). \end{array}$$

### 2.5. Statistical Analysis

The number of sessions required to complete the reversal task was plotted on a Kaplan-Meier curve, and group performance was compared using the Cox proportional-hazard regression model. Due to the effects of heteroscedasticity (collection of random variables) and positive skewing effects, all reversal and rereversal task variable data were log transformed prior to being analysed. Data from the other variables collected for both young and aged and stroke and sham animals were analysed using a two-way analysis of variance (ANOVA), followed by Sidak's multiple comparisons post hoc test. Data from aged sham, aged stroke, and aged stroke+TrkB-Fc animals were analysed with a one-way ANOVA, followed by Sidak's multiple comparisons post hoc test. The Cox proportional hazard regression model was performed using STATA 13.0, with all remaining analyses being performed using Prism 6.0.

### 2.6. Exclusion Criteria

Conducting studies in aged rodents is notoriously difficult, with higher levels of attrition reported poststroke [37]. In the present study, one aged sham animal, one aged stroke+saline animal, one aged stroke+IgG-Fc animal, and three aged stroke+TrkB-Fc animals were sacrificed and excluded from reversal analysis due to attrition/mortality. Two young stroke animals, one aged stroke+saline animal, and one aged stroke+IgG-Fc animal were excluded because they showed no visible infarcts following cresyl violet staining. Furthermore, one aged sham animal, one young sham animal, and one aged stroke+TrkB-Fc animal were excluded from all analysis as they failed to meet the reversal criterion. Another aged stroke+TrkB-Fc animal was excluded due to an unsuccessful hydrogel injection. Furthermore, one aged stroke+saline animal, one aged stroke+IgG-Fc animal, and one aged stroke+TrkB-Fc animal also died during rereversal testing and were excluded from rereversal analysis. The final number of animals per treatment group for the reversal and rereversal tasks are shown in Table 1.

## 3. Results

### 3.1. Infarction Volume Quantification

**Figure 2:**
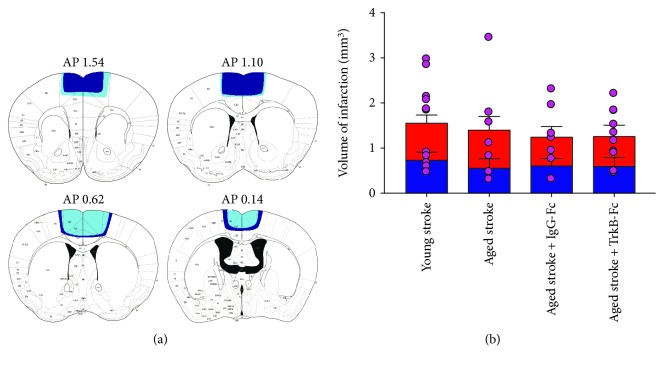
Schematic representation of the mean infarct area in aged (dark blue) and young (light blue) mice following a 22-minute bilateral PFC stroke. Stroke damage extends from 1.54 to 0.14 AP (a). Infarct volume was measured for both the left (blue bar) and right (red bar) hemispheres and for the whole brain (pink dots; b) in young stroke, aged stroke, and aged+TrkB-Fc stroke animals. Data are expressed as mean ± S.E.M. for *n* = 10-14 per group.

Histological assessment of infarct volume was assessed at day 56 poststroke, using cresyl violet staining (Figure 2). Damage following a stroke to the PFC extended into the primary motor cortex (M1), anterior cingulate cortex (ACC), and the supplementary motor cortex (Figure 2(a)). No differences were found in asymmetry between damage to the left and right hemispheres following a bilateral PFC stroke in both young and aged animals treated with saline (young—left: 0.759 ± 0.151 mm^3^, right: 0.822 ± 0.172 mm^3^; aged—left: 0.581 ± 0.172 mm^3^, right: 0.845 ± 0.276 mm^3^; *p* = 0.6816, *F*(1, 32) = 0.1714; Figure 2(b)). Furthermore, no differences were found between the average total stroke volume between these groups (young: 1.58 ± 0.274 mm^3^; aged: 1.38 ± 0.404 mm^3^; *p* = 0.5950, *F*(1, 32) = 0.2883).

Aged stroke animals treated with IgG-Fc or TrkB-Fc showed no asymmetry between left and right hemispheres following a PFC stroke (IgG-Fc—left: 0.632 ± 0.139 mm^3^, right: 0.640 ± 0.205 mm^3^; TrkB-Fc—left: 0.621 ± 0.170 mm^3^, right: 0.665 ± 0.221 mm^3^; *p* = 0.9247, *F*(1, 30) = 0.0090; Figure 2(b)) and presented with average stroke volumes similar to aged and young stroke animals (IgG-Fc—1.28 ± 0.259 mm^3^; TrkB-Fc, 1.29 ± 0.186 mm^3^; *p* = 0.9739, *F*(1, 30) = 0.0011). These data suggest that any behavioural differences seen *in vivo* between young and aged animals and also between aged animals treated with IgG-Fc and TrkB-Fc is unlikely to be due to differences in stroke volume.

### 3.2. Survival Curve of Reversal and Rereversal Tasks

**Figure 3:**
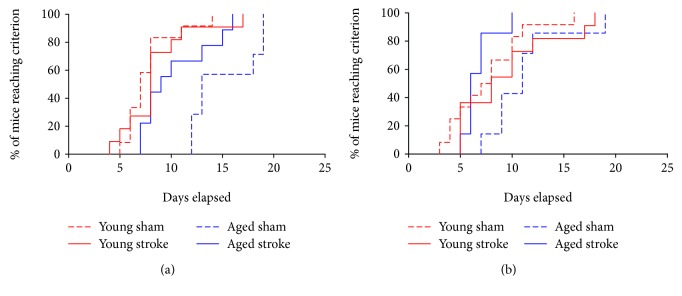
Survival curve of the number of sessions each animal is required to make to reach the criterion (≥80% accuracy across two consecutive days) in the reversal (a) or rereversal (b) tasks. Aged animals (sham—dotted blue line; stroke—solid blue line) take longer to learn both tasks compared to young animals (sham—dotted red line; stroke—solid red line). Aged stroke animals performed better than aged sham controls.

Lesion studies have previously illustrated the involvement of the PFC in behavioural flexibility on the reversal task [20, 22]. Furthermore, the learning capacity of aged animals with stroke to the PFC during VD tasks in the operant touchscreen chambers has yet to be established. Therefore, the Kaplan-Meier survival curves and Cox regression analyses were used to look at the number of days each group needed in order to reach the criterion for the reversal task (assessed at poststroke day 17) and rereversal task (assessed at poststroke day 37) (Figure 3). A test for proportional-hazard assumption showed that both the reversal and rereversal data sets showed no proportionality (data not shown).

A significant interaction effect of age and stroke was seen across the reversal task (*p* < 0.0001; *χ*^2^ = 27.18; *df* = 4; Figure 3(a)). Young sham animals completed the reversal task approximately 7.9 times faster than aged sham animals (hazard ratio (HR): 7.913, *p* = 0.0004, 95% confidence interval (CI): 2.498-25.07). Stroke to the PFC of young animals had no significant effect of learning in the reversal task (HR: 1.348, *p* = 0.3814, CI: 0.592-3.06). Interestingly, stroke to the PFC of aged animals facilitated improved learning in the reversal task, with aged stroke animals completing the task approximately 4.2 times faster than their aged-matched sham counterparts (HR: 4.283, *p* = 0.0272, CI: 2.460-11.221). No significant differences were seen between the performance of young animals and that of aged stroke animals in the reversal task (HR: 1.125, *p* = 0.5936, CI: 0.4662-2.715).

A significant interaction effect of age and stroke was also seen across all animals in the rereversal task (*p* = 0.0007; *χ*^2^ = 15.02; *df* = 4; Figure 3(b)). Young sham animals were found to master the rereversal task approximately 2.4 times faster than aged sham animals; however, this result only trended towards significance (HR: 2.447, *p* = 0.0533, CI: 0.829-5.136). Similar to the performance on the reversal task, there was no significant difference between young sham and young stroke animals on the rereversal task (HR: 1.848, *p* = 0.2075, CI: 0.7112-4.801). However, aged stroke animals were found to outperform their aged-matched sham counterparts, mastering the rereversal task 1.8 times faster (HR: 1.833, *p* = 0.0473, CI: 0.2166-4.006). No significant differences were seen between the performance of young and aged stroke animals in the rereversal task (HR: 1.333, *p* = 0.1293, CI: 0.5169-3.44).

These data indicate that there is an age-related decline in cognitive flexibility in mice. In addition, stroke to the PFC of aged mice appears to reopen a critical window for the improvement of recovery that results in the facilitation of performance across both of these tasks, albeit, to different extents.

### 3.3. Performance in the Reversal Task after PFC Stroke

**Figure 4:**
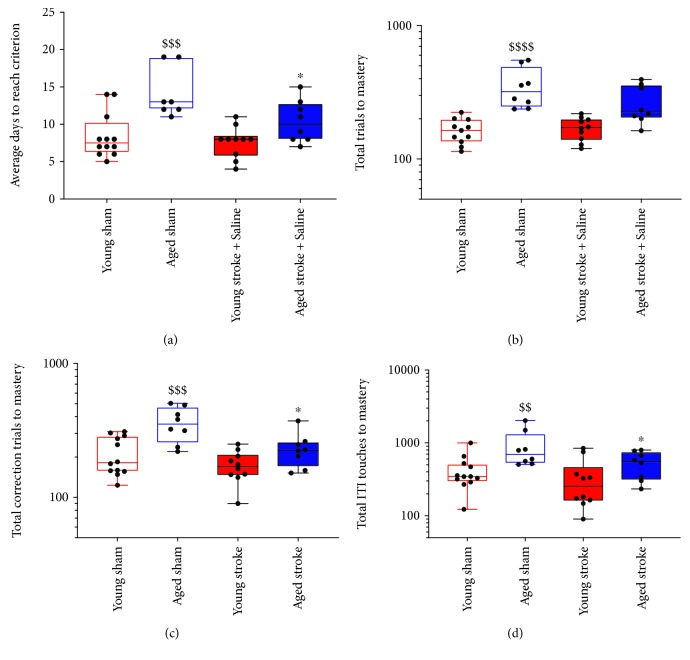
The effect of age (young (red) versus aged (blue)) and PFC stroke (filled boxes) on the number of consecutive days (a), total trials (b), total correction trials (c), and total ITI touches (d) required to master the reversal task compared to sham (open boxes) controls. $$ = *p* < 0.01, $$$ = *p* < 0.001, and $$$$ = *p* < 0.0001 compared to young sham. ^∗^ = *p* < 0.05 compared to aged-matched sham controls.

To investigate the performance of age-matched animals after PFC stroke in the reversal task, a two-way ANOVA was performed on log-transformed data (Figure 4). Throughout the reversal task, an overall effect of age and stroke surgery was seen in the number of consecutive days required to meet the criterion (stroke, *p* = 0.0261, *F*(1, 35) = 5.394; age, *p* < 0.0001, *F*(1, 35) = 25.24; Figure 4(a)), total number of correction trials (stroke, *p* = 0.0054, *F*(1, 35) = 8.784; age, *p* < 0.0001, *F*(1, 35) = 20.49; Figure 4(c)), and total number of ITI touches (stroke, *p* = 0.0367, *F*(1, 35) = 4.71; age, *p* = 0.0015, *F*(1, 35) = 11.82; Figure 4(d)). An age effect but not stroke effect was also observed in the total number of trials required to meet the reversal criterion (stroke, *p* = 0.1546, *F*(1, 35) = 2012; age, *p* < 0.0001, *F*(1, 35) = 36.29; Figure 4(b)). However, no significant interactions were observed between age and stroke effects across all of these measures (consecutive days, *p* = 0.1509, *F*(1, 35) = 2.156; total trials, *p* = 0.0926, *F*(1, 35) = 2.994; total correction trials, *p* = 0.1460, *F*(1, 35) = 2.211; and total ITI touches, *p* = 0.1709, *F*(1, 35) = 1.9151).

Sidak's multiple comparison test confirmed that aged sham animals require significantly more consecutive days (*p* = 0.0003), total trials (*p* < 0.0001), total correction trials (*p* = 0.0008), and total ITI touches (*p* = 0.0090) to make the criterion in the reversal task, relative to young sham animals. Furthermore, no significant differences were seen between young sham and young stroke animals across any of these variables (consecutive days, *p* = 0.9163; total trials, *p* = 0.9969; total correction trials, *p* = 0.6816; and total ITI touches, *p* = 0.9384). However, aged stroke animals were found to require significantly less consecutive days (*p* = 0.0463), total correction trials (*p* = 0.0265), and total ITI touches (*p* = 0.0316) to reach the criterion in the reversal task, relative to aged sham control animals. In contrast, there was no significant difference seen in the number of total trials (*p* = 0.1604) that were required from aged stroke animals to make the reversal criteria relative to aged sham animals. Relative to young stroke animals, aged stroke animals were found to require significantly more total trials to master the reversal task (*p* = 0.0217); however, no other differences were observed between these two groups across the other variables (consecutive days, *p* = 0.764; total correction trials, *p* = 0.1597; and total ITI touches, *p* = 0.4730). Moreover, aged stroke animals required significantly more consecutive days to master the reversal task compared to young sham animals (*p* = 0.0403), but no differences were seen in the number of total trials (*p* = 0.1136), total correction trials (*p* = 0.4319), or total ITI touches (*p* = 0.2664).

Together these data support an age-related reduction of performance across the reversal task in aged sham mice. Moreover, stroke to aged mice appears to enhance reversal learning, an effect not observed in young mice.

### 3.4. Performance in the Rereversal Task after PFC Stroke

**Figure 5:**
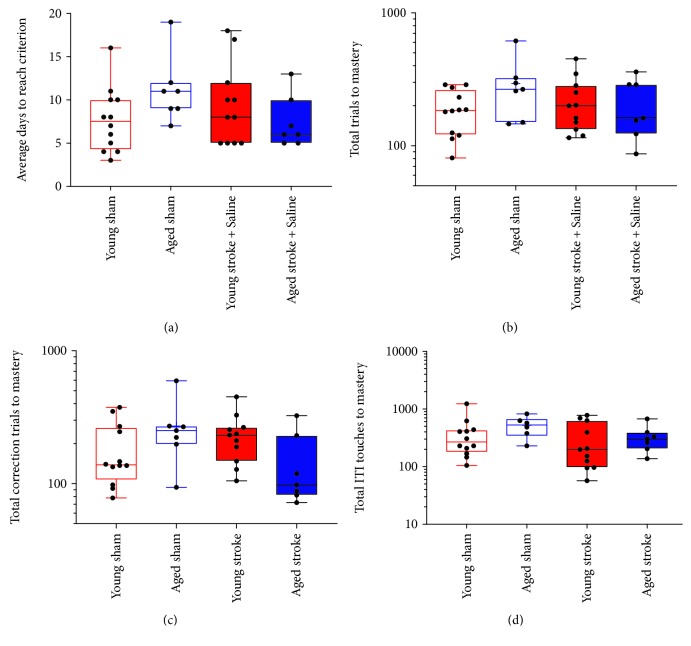
The effect of age (young (red) versus aged (blue)) and PFC stroke (filled boxes) on the number of consecutive days (a), total trials (b), total correction trials (c), and total ITI touches (D) required to master the rereversal task compared to sham (open boxes) controls.

To investigate the performance of age-matched animals after stroke to the PFC in the rereversal task, a two-way ANOVA was conducted on log-transformed data collected from day 37 poststroke (Figure 5). Assessment of the rereversal task revealed a greater variability in the data compared to the reversal task data. No effect of either age or stroke surgery was observed in the number of consecutive days (stroke, *p* = 0.4563, *F*(1, 33) = 0.5853; age, *p* = 0.5686, *F*(1, 33) = 0.3317; Figure 5(a)), total trials (stroke, *p* = 0.3358, *F*(1, 33) = 0.953; age, *p* = 0.9121, *F*(1, 33) = 0.0123; Figure 5(b)), and total ITI touches (stroke, *p* = 0.1694, *F*(1, 33) = 1.976; age, *p* = 0.3378, *F*(1, 33) = 0.800; Figure 5(d)). However, a significant effect of age but not stroke was seen in the total number of correction trials required to make the reversal criterion (stroke, *p* = 0.4252, *F*(1, 33) = 0.652; age, *p* < 0.0140, *F*(1, 33) = 7.734; Figure 5(c)). In addition, a significant interaction between age and stroke surgery was only seen in the total number of trials required to make the criterion (total trials, *p* = 0.0288, *F*(1, 33) = 5.253; consecutive days, *p* = 0.0513, *F*(1, 33) = 4.089; total correction trials, *p* = 0.9596, *F*(1, 33) = 0.0263; total ITIs, *p* = 0.4912, *F*(1, 33) = 0.4849).

Sidak's multiple comparison test failed to find a significant difference between the performance of young and aged sham animals in the rereversal task (consecutive days, *p* = 0.2679; total trials, *p* = 0.3497; total correction trials, *p* = 0.2851; and total ITI touches, *p* = 0.6882). Furthermore, no significant differences were found between young sham and young stroke animals (consecutive days, *p* = 0.7331; total trials, *p* = 0.7229; total correction trials, *p* = 0.9267; and total ITI touches, *p* = 0.9341) or between aged sham and stroke animals (consecutive days, *p* = 0.3097; total trials, *p* = 0.1738; total correction trials, *p* = 0.9473; and total ITI touches, *p* = 5610). Lastly, aged stroke animals performed similarly to both young sham (consecutive days, *p* = 0 to >0.9999; total trials, *p* > 0.9999; total correction trials, *p* = 0.8266; and total ITI touches, *p* = 0.5451) and young stroke animals (consecutive days, *p* = 0.7297; total trials, *p* = 0.1738; total correction trials, *p* = 0.2664; and total ITI touches, *p* = 0.9889) in the rereversal task.

### 3.5. TrkB-Fc Blocks the Stroke-Induced Improvement in Performance

**Figure 6:**
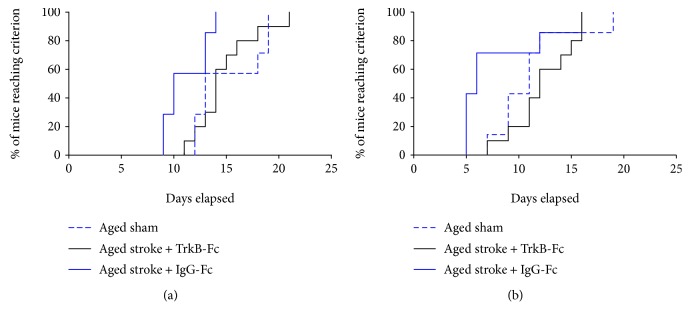
Survival curve of the number of sessions each animal is required to make to reach the criterion (≥80% accuracy across two consecutive days) in the reversal (a) or rereversal (b) tasks. Aged stroke animals (solid blue line) take longer to learn both tasks compared to aged sham controls (dotted blue line). Aged stroke animals performed better than aged sham controls. This effect was blocked following the administration of the BDNF decoy, TrkB-Fc (solid black line).

To investigate the involvement of BDNF signalling in reversal and rereversal learning after PFC stroke, performance was compared between aged sham animals and aged stroke animals treated with either IgG-Fc or the BDNF scavenger, TrkB-Fc (Figure 6). A Cox regression analysis of the Kaplan-Meier curves revealed a significant treatment effect (*p* = 0.0124, *χ*^2^ = 9.16, *df* = 3; Figure 6(a)). Specifically, aged stroke animals treated with IgG-Fc completed the reversal task approximately 1.3 times faster than aged sham animals (HR: 1.310, *p* = 0.0272, CI: 0.4369-3.684). Moreover, aged stroke animals treated with TrkB-Fc completed the reversal task approximately 3.1 times slower than aged stroke control animals (HR: 3.136, *p* = 0.0073, CI: 0.8256-11.91), performing similarly to aged sham animals (HR: 0.9144, *p* = 0.8758, CI: 0.297-2.811).

**Figure 7:**
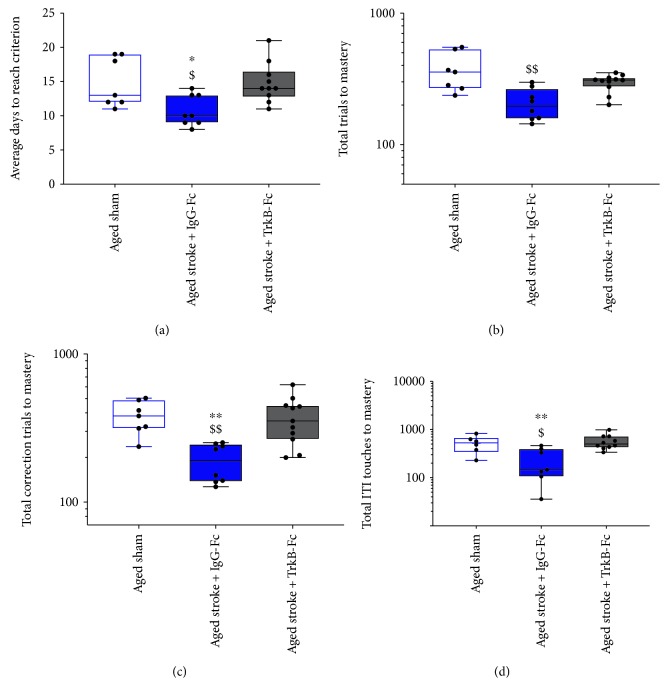
The effect of PFC stroke and TrkB-Fc treatment on the number of consecutive days (a), total trials (b), total correction trials (c), and total ITI touches (d) required to master the reversal task. Aged sham: nonfilled blue box; aged stroke+IgG-Fc (hydrogel control): filled blue box; aged stroke+TrkB-Fc: filled grey box. $ = *p* < 0.05 and $$ = *p* < 0.01 compared to aged sham. ^∗^ = *p* < 0.05 and ^∗∗^ = *p* < 0.01 compared to TrkB-Fc-treated aged stroke animals.

A one-way ANOVA was performed on log-transformed data from all animals, and it confirmed a significant treatment effect for the number of consecutive days (*p* = 0.0144, *F*(2, 23) = 5.174; Figure 7(a)), total number of trials (*p* = 0.0019, *F*(2, 23) = 8.346; Figure 7(b)), total number of correction trials (*p* = 0.0015, *F*(2, 23) = 8.757; Figure 7(c)), and total number of ITI touches (*p* = 0.0201, *F*(2, 23) = 4.656; Figure 7(d)) to reach the criterion on the reversal task. Sidak's multiple comparison test further revealed that aged stroke animals treated with IgG-Fc required significantly less consecutive days (*p* = 0.0346), total trials (*p* = 0.0013), total correction trials (*p* = 0.0048), and total ITI touches (*p* = 0.0184) compared to aged sham animals. TrkB-Fc treatment was found to block this stroke-induced effect, with TrkB-Fc-treated aged stroke animals requiring significantly more consecutive days (*p* = 0.0222) and correction trials (*p* = 0.0029) to reach the criterion relative to aged stroke control animals. No significant differences were observed in the number of total trials (*p* = 0.0538) or total ITIs (*p* = 0.0987) between stroke+TrkB-Fc-treated animals and aged stroke controls on the reversal criterion. Finally, TrkB-Fc-treated aged stroke animals performed the same as aged sham animals across all variables (consecutive days, *p* = 0.9992; total trials, *p* = 0.1405; total correction trials, *p* = 0.9798; and total ITI touches, *p* = 0.5304).

Analysis of the rereversal task revealed a significant effect of treatment across all animals as detected using a Cox regression analysis of the Kaplan-Meier survival curves (*p* = 0.0473, *χ*^2^ = 7.67, *df* = 3; Figure 6(b)). Aged stroke animals treated with IgG-Fc completed the rereversal task similarly to aged sham animals (HR: 1.212, *p* = 0.0831, CI: 0.316-3.225). However, aged stroke animals treated with TrkB-Fc completed the rereversal task approximately 3.9 times slower than aged stroke control animals (HR: 3.911, *p* = 0.0012, CI: 0.722-6.014), with the performance of the TrkB-Fc-treated animals being similar to aged sham controls (HR: 1.142, *p* = 8208, CI: 0.363-3.59).

**Figure 8:**
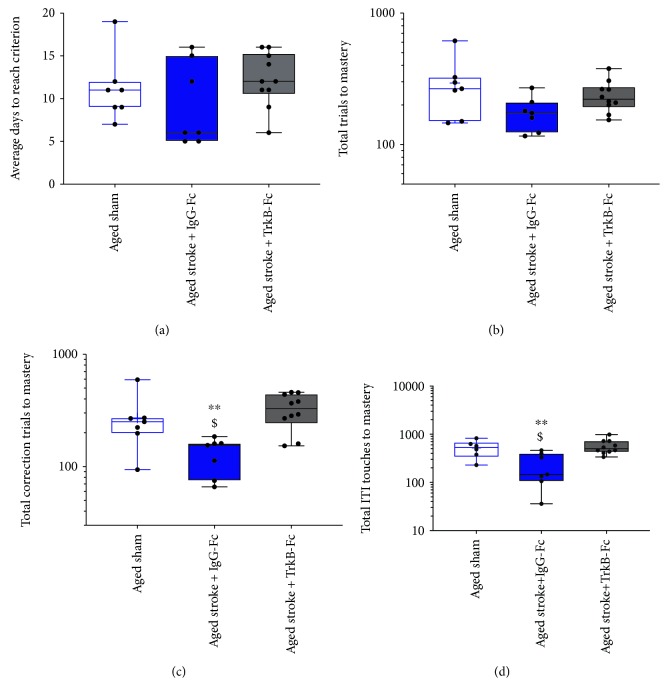
The effect of PFC stroke and TrkB-Fc treatment on the number of consecutive days (a), total trials (b), total correction trials (c), and total ITI touches (d) required to master the rereversal task. Aged sham: nonfilled blue box; aged stroke: filled blue box; aged stroke+TrkB-Fc: filled grey box. $ = *p* < 0.05, $$ = *p* < 0.01, and $$$ = *p* < 0.001 compared to aged sham. ^∗^ = *p* < 0.05 compared to TrkB-Fc-treated aged stroke animals.

A one-way ANOVA was performed on log-transformed rereversal data, which confirmed a significant treatment effect on the total number of correction trials (*p* = 0.0117, *F*(2, 21) = 5.598; Figure 8(c)) and ITI touches (*p* = 0.0049, *F*(2, 21) = 7.008; Figure 8(d)) but not on the number of consecutive days (*p* = 0.3397, *F*(2, 21) = 0.3397; Figure 8(a)) or the total number of trials (*p* = 0.1154, *F*(2, 21) = 2.41; Figure 8(b)). In addition, Sidak's multiple comparisons revealed that aged stroke mice treated with IgG-Fc required significantly more total correction trials (*p* = 0.0465) and ITI touches (*p* = 0.0315) to make a rereversal compared to aged sham animals. However, no significant difference was seen in the number of consecutive days (*p* = 0.6556) or total number of trials (*p* = 0.0426) between these animals. Treatment with TrkB-Fc was shown to dampen the stroke-induced effect, with TrkB-Fc-treated stroke animals performing similarly to aged sham animals across all variables (consecutive days, *p* = 0.8495; total trials, *p* = 0.5158; total correction trials, *p* = 0.5119; and total ITI touches, *p* = 0.8860). Conversely, TrkB-Fc-treated aged stroke animals required significantly more total correction trials (*p* = 0.0091) and total ITI touches (*p* = 0.0049) to complete the rereversal task compared to aged stroke animals. However, no difference was observed between these treatment groups in regard to the number of consecutive days (*p* = 0.3085) or total trials taken (*p* = 0.4114).

## 4. Discussion

In recent years, touchscreen-based cognitive testing has been designed to target components of human-based cognition in rodents, thereby maximising the translational potential of preclinical experiments [18, 19, 21]. In the present study, we used this technology to demonstrate an age-related decline in cognitive flexibility on the VD task in mice. In addition, we showed that stroke to the PFC had no effect on reversal learning in young mice, but facilitated an improvement in learning in aged mice. Finally, we show that this stroke-induced improvement in learning observed in the aged mice was BDNF dependent, as the improvement in learning was blocked following administration of the BDNF decoy, TrkB-Fc.

The PFC is a region of the brain that is heavily involved with complex cognitive processes, such as behavioural flexibility [22]. Extensive evidence demonstrates that set-shifting performance is critically dependent on the dorsolateral prefrontal cortex (PFC) in primates or the medial prefrontal cortex (mPFC), which is the rodent homolog [38, 39]. Age-related alterations in both the architecture and molecular composition of the PFC are known to contribute to cognitive decline seen in healthy aged animals [40, 41]. Consistent with this, our study revealed an age-related decline in VD reversal learning, with aged sham animals requiring more consecutive days, trials, correction trials, and ITI touches to reach the criterion compared to young sham animals. This finding is supported by human, primate, and rodent reversal studies that have reported cognitive slowing in aged cohorts using other cognitive assessments [40–43]. Moreover, this age-related cognitive slowing is not only applicable to behavioural flexibility but other cognitive domains such as spatial memory, attention, and working memory [43, 44]. Nonetheless, it is important to emphasise that these aged mice could still demonstrate the same degree of cognitive flexibility as young mice; however, they just required a longer period to reach this potential. It is also interesting to note that this age-related effect appears to be dampened in the rereversal task where the animals switch their learning back to the original stimuli pairing, indicating that the ability to adapt to previously learnt tasks remains intact in aged animals. The young versus age difference we report highlights the need to assess both young and aged cohorts as they respond differently to strokes, including eliciting a different molecular/transcriptional response [31, 45] and therefore potentially requiring different pharmacological and or physical therapies to enhance recovery.

In contrast to previous reports [22, 23], our findings showed that a stroke to the PFC had no effect on VD reversal and rereversal learning in young mice, yet it improved performance in aged mice. There are a few possible explanations for these observations. Clinical studies have revealed that whilst unilateral lesions to the PFC fail to affect performance on a VD reversal task, bilateral lesions to the PFC cause severe impairments in this task [46]. This finding suggests that a larger lesion involving both cortices is required to create this deficit in cognitive flexibility in humans, which is also supported by reports of dementia in patients with global vascular impairment [47]. A second explanation for a lack of stroke-induced impairment seen in the present study may lie in the neural circuitry that has been proposed to control cognitive flexibility. Lesion and stroke studies utilising touchscreen-based VD reversal tasks have highlighted the involvement of the mPFC, orbitofrontal cortex (OFC), and dorsolateral striatum in facilitating performance [8, 9, 48, 49], whereas the ventromedial PFC (vmPFC) and basolateral amygdala (BLA) play a role in restricting performance [48, 49]. It is possible then, that the site of the PFC strokes in the present experiment may have played an inhibitory role in reversal learning, similar to the BLA or vmPFC. In addition, we highlight that the age-associated improvement in cognitive flexibility is associated with an elevation in BDNF levels.

Human and rodent studies have both illustrated that younger brains recover more effectively than aged brains [50–52], possibly due to altered genetic and cellular responses observed in aged animals [53]. Whilst age-related differences in infarct volume have been investigated, these findings remain inconclusive [45, 54, 55], and age-related differences in infarct expansion and resolution and changes in peri-infarct plasticity remain poorly understood. Furthermore, as the current study observed similar infarct volumes between young and aged animals, we propose that the remaining neurons in nuclei found in peri-infarct regions in young mice are likely to have a greater functional reserve, relative to old animals. The volume of the PFC has been reported to decrease with age, which could mean that the proportion of the PFC affected in the current stroke model may have been more detrimental to aged animals [56]. However, the exact function of this age-related loss of volume remains debated, with aged brains presenting with changes to dendritic arbour and sprouting profiles that have been proposed to help maintain the same number of synapses throughout the brain [57], thereby compensating for any cell death [58]. It is possible that the differences in functional reserve is solely plasticity mediated and dependent on changes in BDNF levels. However, much needed research is still needed to confirm these hypotheses.

The BDNF-mediated facilitation of reversal learning/cognitive flexibility that was observed in the current study may reflect a reopening of a critical window for functional recovery of cognition after stroke. Whilst the therapeutic potential of BDNF has been illustrated in preclinical stroke models [27–29], clinical success has been challenged by poor blood-brain barrier (BBB) permeability, short half-life, and off-target effects. However, with recent advances in technology, novel biomaterial drug-delivery systems offer the ability to circumvent these issues and provide regenerative agents directly to the injured brain. With this in mind, studies are underway assessing VD reversal task learning in aged stroke animals that receive recombinant human BDNF (rhBDNF) via a hydrogel implanted into the stroke cavity. We hope this may be able to “tap into” this BDNF-mediated mechanism to further promote recovery after stroke. It has also been postulated that a combinational treatment may be required to provide functional recovery in stroke patients especially in aged populations. Supporting this, Clarkson et al. have reported that aged mice receiving motor cortex strokes required treatment with BDNF and a BDNF-inducing AMPAkine in order to gain the same level of functional recovery seen in young mice receiving BDNF treatment alone [28]. Future work in our lab hopes to investigate the therapeutic potential of this combinational approach, as this highlights the need to not only assess changes in BDNF but also the need to assess the role of AMPA receptor-mediated plasticity [28].

The lack of stroke-induced impairment seen in young mice is a limitation of the current study; however, this finding does match the clinical presentation of cognitive symptoms that are seen in humans following stroke. Cognitive impairments in elderly stroke patients are well documented; however, very few studies have reported these impairments in younger patients [55]. To improve our stroke model, future work could investigate the effect of focal lesions in Cerebral Autosomal Dominant Arteriopathy with Subcortical Infarcts and Leukoencephalopathy (CADASIL) mice. These genetically modified mice present with an abnormal regulation of blood flow and neurovascular dysfunction, which is known to be an underlying cause for vascular cognitive impairment [59]. In light of this, we would expect our focal lesion models to have a more pronounced effect on cognitive flexibility in young mice, which may result in a detectable impairment in reversal learning.

The brain is known to undergo significant functional reorganisation in response to motor learning in the reaching task, and subsequent motor maps expand into neighbouring regions after stroke and brain injury, a process known as plasticity [60, 61]. Functional recovery resulting from this reorganisation has also been found to be dependent on BDNF signalling [27–30]. BDNF, like most neurotrophins, plays a critical role in neuronal development, differentiation, and survival. In addition, BDNF plays a central role in modulating components of synaptic plasticity both during development and throughout adulthood [62]. Such functions are mainly mediated via the release of BDNF that is regulated by neuronal activity [62, 63]. Collectively, these findings support the idea that BDNF is likely to play a role during the reparative phase after stroke, a period where the brain repairs itself to try compensate for the damaged tissue. Further support comes from clinical studies that have shown that aerobic exercise is sufficient to facilitate cognitive recovery (including cognitive flexibility) [64], an effect that is thought to be somewhat mediated by elevations in BDNF levels [65]. In conjunction with our study, these findings support the potential of BDNF to facilitate recovery of cognitive function in humans, not only after stroke but also with normal ageing.

BDNF is believed to have a beneficial effect on stroke recovery via several mechanisms: increased angiogenesis [66] and neurogenesis [67], increased brain repair [68], and enhanced synaptic plasticity [27, 69]. It is reasonable to predict that age-related changes in the BDNF-TrkB signalling pathway may have altered the responsiveness of the peri-infarct tissue to these mechanisms of plasticity in the aged mice of the current study. However, whether or not the expression of BDNF and its high-affinity receptor, TrkB, is reduced or increased in aged rodents remains a matter of debate [28, 70, 71]. We are currently investigating age-related changes in neurotrophin signalling pathways and treatments in the hope to better understand the functional role of BDNF throughout stroke-induced cognitive impairments. Because BDNF is associated with cognitive flexibility [48, 72], treatment with this trophic factor or other pharmacological agents that stimulate an increase in BDNF expression may be an effective therapy in alleviating slowing cognitive flexibility in other models of cognitive impairment.

The touchscreens require mice to be tested daily for an extended period of time in order to learn the task and then to perform both the reversal and rereversal tasks poststroke. Previous experiments have reported that PFC BDNF levels have been found to remain stable whilst rodents undergo classical learning, memory, and/or extinction tasks [73, 74]. These findings indicate that the learning experiences alone are not enough to significantly affect PFC BDNF expression. Therefore, the most parsimonious explanation for the stroke-induced improvement in cognitive flexibility observed in the current study in aged mice is that BDNF levels are elevated as a result of the stroke, which also fits with TrkB-Fc's ability to dampen this response.

Overall, this study suggests that aged animals still have the capacity to learn and undertake complex cognitive tasks using an operant-based touchscreen, allowing the touchscreens to be used to investigate impairments and interventions in cognitive disorders. Furthermore, stroke to the PFC of young animals has no effect on cognitive flexibility, whereas stroke to the PFC of aged animals resulted in a significant improvement in performance in both the reversal and rereversal tasks. In addition, aged stroke mice treated with the BDNF decoy, TrkB-Fc, blocked the stroke-induced improvement in cognitive flexibility. Finally, we demonstrate that stroke to the PFC of aged mice reopens a critical window for functional recovery that is BDNF dependent.

## Figures and Tables

**Table 1:** Summary showing treatment group sizes included for analysis at the start of the experiment and for both the reversal and rereversal tasks. Animal numbers that were excluded for both the reversal and rereversal tasks are shown in brackets.

Treatment group	Starting **n**	Final **n** for reversal	Final **n** for rereversal
Young sham	13	12 (1)	12 (1)
Young stroke	13	11 (2)	11 (2)
Aged sham	9	7 (2)	7 (2)
Aged stroke	10	8 (2)	7 (3)
Aged stroke+IgG-Fc	10	8 (2)	7 (3)
Aged stroke+TrkB-Fc	16	11 (5)	10 (6)

## Data Availability

All data used in the current manuscript have been placed in a repository at the University of Otago.
